# Supplementary material for: Resilience amid chaos: The role of Gaza medical points
Source: PLOS Glob Public Health. 2025 Sep 17;5(9):e0005073. doi: 10.1371/journal.pgph.0005073 (PMC12443234; doi:10.1371/journal.pgph.0005073)
Supplement: Attachment — Submitted filename: reviewer-notes.docx [file pgph.0005073.s002.docx]

Thank you for this important and timely piece of work amidst a very trying time in Gaza. This study provides valuable insights into the role of medical points in Gaza amid the ongoing Israeli assault. While the research is well-structured and addresses a critical issue, several areas require refinement. Please see comments below for breakdown by section:

**Introduction**

- Please ensure to have relevant citations early (ex: line 64; this study is not cited until the end of the paragraph); furthermore
- Please ensure that acronyms are spelled out first prior to using them (ex: line 64, the use of *CMAJ*).
- Please have a citation for your statement that the healthcare system is on the brink of collapse
  - I suggest using the UN report published in October 2024 regarding attacks on healthcare
- Very clear definition of medical points; might be good to have a citation pointing to their use in other settings previously
- With regards to disruption of vaccination services, may also be good to point to literature where war was seen as driver of drop off of vaccination (there is a study done in Yemen that highlights this). This may strengthen your assertion that the war is a major driver in the vaccination rates

**Methodology**

- Citation needed for Google Forms
- Was participant data anonymized?
- Please clarify if the data for each MP was extracted via one representative or if multiple workers within the MPs were able to fill out the surveys
- Please clarify how you were able to qualify “failure to respond was due to internet connectivity” versus other reasons for non-response
  - This may also need a citation regarding availability of internet connectivity in the Gaza Strip during the study period
- Please clarify what literature was reviewed to develop the survey
- Please clarify how the scale of drug availability was determined; i.e. what does “fully available” mean, and how was this definition communicated to those who filled out the survey?

**Results**

- How many total MPs were reached out to for the study? You will need this for the denominator to see response rate.
- Please report the results consistently as follows: n (%)
- Please ensure the data is reported objectively, i.e. with numbers as opposed to with qualifiers such as “significant”; this may be done in the discussion
- Please clarify if in line 181 n=4 refers to the number of workers harmed or arrested, or if this refers to the number of MPs that had at least one worker harmed or arrested
- In line 183, please change “targeted” to “attacked”
- Definitions within the survey (ex: electricity availability in line 212-213), should be in the methods

**Discussion**

- The study in reference 7 appears to focus on the elderly, yet the statement is made more generally; please ensure other references that can speak more generally to this statement or revise the statement to reflect the population studied in the relevant citation
- There are statements within the discussion which overstate the results or make claims that are not within the scope of the study, such as in line 254 where you speak about shortage of medications exacerbating the health crisis.
  - This occurs in line 275 where the findings of your study are generalized to the entire Gaza strip and is then compared to Yemen. It would be more appropriate to state that the medication shortage is observed within MPs, since hospitals and other static structures within Gaza are not included within the study.
- The discussion restates the findings of the study; this space should be used to discuss recommendations and make references to the literature about the state of healthcare in other conflict settings if literature on medical points is limited
- It may be useful to speak to the blockade that may affect the influx of critical medications and equipment in the Gaza Strip

**Limitations**

- This section can be expanded to include response rate, that the study only looked at MPs and its results may not be reflective of all medical structures in Gaza, among others

**Conclusion**

- Please specify “urgent action”
