# Supplementary material for: Resilience amid chaos: The role of Gaza medical points
Source: PLOS Glob Public Health. 2025 Sep 17;5(9):e0005073. doi: 10.1371/journal.pgph.0005073 (PMC12443234; doi:10.1371/journal.pgph.0005073)
Supplement: Attachment — Submitted filename: Response to Reviewers Comments.docx [file pgph.0005073.s004.docx]

**Introduction**:

**1&2. Reviewer’s Comment:**

“Please ensure to have relevant citations early (ex: line 64; this study is not cited until the end of the paragraph); furthermore Please ensure that acronyms are spelled out first prior to using them (ex: line 64, the use of CMAJ).”

**Response:**
Thank you for your helpful comment. We have revised the paragraph to introduce the citation earlier and have spelled out the acronym CMAJ at first mention to enhance clarity and consistency. The revised sentence now reads:

"A 2009 study published in the *Canadian Medical Association Journal (CMAJ) (1)* highlighted the pre-existing strain on Gaza’s healthcare infrastructure even before the 2008–2009 war."

3. **Reviewer’s Comment:**

"Please have a citation for your statement that the healthcare system is on the brink of collapse. I suggest using the UN report published in October 2024 regarding attacks on healthcare."

**Response:**
Thank you for this valuable suggestion. We have revised the paragraph to include a citation from the October 2024 report by the Office of the High Commissioner for Human Rights (OHCHR), which documents the severe deterioration of the healthcare system in Gaza due to sustained attacks. The revised text now reads:

“The ongoing 2023 military assault on Gaza has had devastating effects on the district’s medical infrastructure, resources, and personnel, pushing the healthcare system to the brink of collapse *as documented by the Office of the High Commissioner for Human Rights (OHCHR) (2). “*

4. **Reviewer’s Comment:**

"Very clear definition of medical points; might be good to have a citation pointing to their use in other settings previously."

**Response:**

Thank you for this thoughtful suggestion. Indeed, we conducted an extensive literature search; however, we found that the term "medical points" is not consistently used in the same context or with the definition we provide—namely, temporary healthcare units operating within active war zones. For this reason, we initially did not include citations. Nevertheless, in response to your valuable comment, we have now added references to relevant healthcare adaptations from other conflict settings, such as Yemen, Ukraine, and Syria. While these models differ from ours, they help contextualize the concept and underscore the uniqueness of our study. Accordingly, we removed the previous concluding sentence of the Introduction (“Therefore, understanding the role, challenges, and needs of medical points is essential to explore potential strategies for sustainable healthcare delivery in conflict zones like Gaza”) and replaced it with the following new paragraph that incorporates these citations:

*“For all the reasons outlined above, a thorough understanding of the role, challenges, and needs of medical points is critical to informing sustainable healthcare strategies in conflict settings like Gaza. While previous literature has documented healthcare adaptations in other conflict zones, a detailed account of the medical point (MP) model is absent. In Yemen, mobile medical teams were deployed to manage cholera and acute watery diarrhea, and rehydration points were established within public healthcare facilities to address dehydration cases. In Ukraine, MPs were set up at border crossings with Poland to provide first aid and emergency care to fleeing civilians. In Syria, repeated airstrikes prompted the establishment of multiple smaller medical points instead of rebuilding large hospitals, aiming to enhance resilience and avoid total service disruption with single airstrikes. However, none of these studies addressed the operational status, functionality, or resource availability of MPs. This gap in the literature underscores the novelty and necessity of our study.”*

5. ***Reviewer’s Comment:***

“With regards to disruption of vaccination services, may also be good to point to literature where war was seen as driver of drop off of vaccination (there is a study done in Yemen that highlights this). This may strengthen your assertion that the war is a major driver in the vaccination rates.”

**Response:**
Thank you for this insightful suggestion. We have now incorporated supporting evidence from the conflict in Yemen, specifically referencing Camacho et al. (2018), to strengthen our assertion that war is a major driver of declining vaccination coverage and the resurgence of vaccine-preventable diseases. Accordingly, we added the following sentence at the end of the paragraph beginning with “Before the ongoing 2023 war” in the Introduction:
“Similar patterns have been observed in other conflict zones, such as Yemen. Camacho et al. (9) demonstrated that sustained violence and systemic disruptions led to marked declines in vaccination coverage and the resurgence of vaccine-preventable diseases such as measles and cholera.”

**Methodology**

1. **Reviewer’s Comment:**

“Citation needed for Google Forms”

**Response:**
Please find the link below, which will be added to our citations:

<https://docs.google.com/forms/d/e/1FAIpQLScor29Z4TZWql-Rs0w98y0PaRvtiB3tRSaKoXFRCRFwPSvHgg/viewform>

Of note, Google Forms was selected due to its accessibility and user-friendly interface, which facilitated efficient data gathering. The following reference will be added to our citations as well:

Mondal H, Mondal S, Ghosal T, Mondal S. Using Google forms for medical survey: A technical note. Int J Clin Exp Physiol. 2018;5(4):216-8.

2. **Reviewer’s Comment:**

“Was participant data anonymized?”

**Response:**

All participants responded anonymously. The survey did not collect personal identifiers (names or contact information), ensuring confidentiality and protecting the safety of respondents in this conflict setting. – This was added to our methodology at line 179.

3. **Reviewer’s Comment:**

“Please clarify if the data for each MP was extracted via one representative or if multiple workers within the MPs were able to fill out the surveys”

**Response:**

Each medical point provided a single response to the survey, completed by a senior healthcare worker on behalf of that facility.—this was added at line 181

4. **Reviewer’s Comment:**

“Please clarify how you were able to qualify “failure to respond was due to internet connectivity” versus other reasons for non-response

This may also need a citation regarding availability of internet connectivity in the Gaza Strip during the study period”

**Response:**

We noted that some non-responses were likely due to internet and electricity outages. During the study period, Gaza experienced severe communications disruptions – frequent internet blackouts and infrastructure damage often left communities cut off from communication. We attempted follow-up calls where possible, but connectivity issues impeded several medical points from submitting the form**.**

5. **Reviewer’s Comment:**

“Please clarify what literature was reviewed to develop the survey “

**Response:**

The questionnaire was developed after a literature review of health facility assessments in conflict settings. The following paragraph was added to methodology at line 162

“We modelled many questions on the established WHO emergency health resource evaluation framework. The WHO Resources and Services Availability Monitoring System (HeRAMS) gathers information on essential health resources and services in crises [14]. The content covered infrastructure, safety, equipment, drug availability, and staffing, aligning with domains identified in previous field assessments. “

6. **Reviewer’s Comment:**

“Please clarify how the scale of drug availability was determined; i.e. what does “fully available” mean, and how was this definition communicated to those who filled out the survey?”

**Response:**

We added this to our methodology section at line 180:

“ We asked respondents to rate each drug category’s availability from 1 to 5, where one meant ‘not available’ (no drugs in that category on hand) and five meant ‘fully available’ (all essential drugs in that category are on hand). A score of 5 thus indicated the drug category was entirely in stock. This scale was developed for the study to quantify shortages, with intermediate values (2–4) representing partial availability as described in the survey form”

**Results**

1. **Reviewer’s Comment:**

“How many total MPs were reached out to for the study? You will need this for the denominator to see the response rate.”

**Response:**

We added the following sentence to the results section (line 211):

“The response rate was 28/44 (63.6%). “

2. **Reviewer’s Comment:**

“Please report the results consistently as follows: n (%)”

**Response:**

The results section was reviewed and all values were corrected.

3. **Reviewer’s Comment:**

“Please ensure the data is reported objectively, i.e. with numbers as opposed to with qualifiers such as “significant”; this may be done in the discussion”

**Response:**

We agree with the reviewer and the relevant terms were modified in the results section.

4. **Reviewer’s Comment:**

“Please clarify if in line 181 n=4 refers to the number of workers harmed or arrested, or if this refers to the number of MPs that had at least one worker harmed or arrested”

**Response:**

We adjusted the sentence to reflect our true finding: “Four out of 28 medical points (14.3%) reported at least one staff member being injured, arrested, or killed during the period.”

5. **Reviewer’s Comment:**

“In line 183, please change “targeted” to “attacked””

**Response:**

Thank you, and this was changed.

6. **Reviewer’s Comment:**

“Definitions within the survey (ex: electricity availability in line 212-213), should be in the methods”

**Response:**

Thank you, this was added to our methodology:

“Infrastructure and utilities were evaluated with a five-point Likert scale reproduced verbatim from the questionnaire (lines 212–213). For electricity and internet, respondents selected 1 = not available (0 % of the time), 2 = rarely available (< 10 %), 3 = sometimes available (10–50 %), 4 = usually available (> 50 %), or 5 = available all the time (≈ 100 %). The same anchors were applied to psychosocial-support availability, where 1 indicated no support and 5 indicated comprehensive, continuous support. In addition, yes/no items captured the presence of functioning sewage drainage, hand-washing facilities (soap or alcohol-based rub), solid-waste disposal, and a refrigerator suitable for temperature-sensitive medications or vaccines. “

**Discussion**:

1. **Reviewer’s Comment:**

“The study in reference 7 appears to focus on the elderly, yet the statement is made more generally; please ensure other references that can speak more generally to this statement or revise the statement to reflect the population studied in the relevant citation.”

**Response:**
Thank you for this observation. We conducted an extensive search for more general population-based studies assessing access and affordability of healthcare in Gaza prior to the 2023 escalation but were unable to identify suitable peer-reviewed sources with robust population-level data. Accordingly, we revised the sentence to explicitly state that the referenced study pertains to the elderly population. The revised sentence now reads:

*“Prior to this escalation, access to and affordability of healthcare services were significant challenges, with 70% of participants in a 2023 study focused on the elderly population reporting difficulties in these areas.”*

This change ensures accurate representation of the cited study and addresses your concern.

2. **Reviewer’s Comment:**

“There are statements within the discussion which overstate the results or make claims that are not within the scope of the study, such as in line 254 where you speak about shortage of medications exacerbating the health crisis.”

**Response:**
Thank you for this important observation. We agree that the original phrasing may have implied a causal relationship beyond the scope of our data. Accordingly, we revised the sentence to avoid overstating our findings while still contextualizing them within the broader public health situation in Gaza. The revised sentence now reads:

*“This shortage is occurring in a context where Gaza is already experiencing a critical health crisis among children under 5 years, characterized by a significant rise in infectious diseases, including acute respiratory infections, diarrhea, scabies, and jaundice.”*

This revision maintains the relevance of our findings while aligning with the scope and limitations of the study.

3. **Reviewer’s Comment:**

"This occurs in line 275 where the findings of your study are generalized to the entire Gaza Strip and is then compared to Yemen. It would be more appropriate to state that the medication shortage is observed within MPs, since hospitals and other static structures within Gaza are not included within the study."

**Response:**
We acknowledge that our original phrasing may have inadvertently generalized our findings beyond the scope of our study. To address this, we adopted a broader contextual approach: we included available information from UNRWA regarding anticipated medication depletion at its health facilities, while noting the absence of quantified data from other healthcare structures—prior to making any comparison with Yemen. The revised sentence now reads:

*“To the best of our search, we did not find published data quantifying the extent of medication shortages in Gaza’s hospitals, field hospitals, or primary healthcare centers. However, the situation may be comparably severe, as UNRWA warned in December 2024 that at least 60 medications would be depleted at its health facilities by year’s end. Therefore, the overall medication shortage in Gaza may exceed that reported in other conflict zones, such as Yemen, where approximately half of medications were available.”*

This revision preserves the comparative context while clarifying the limits of our dataset and incorporating relevant evidence.

4. **Reviewer’s Comment:**

“The discussion restates the findings of the study; this space should be used to discuss recommendations and make references to the literature about the state of healthcare in other conflict settings if literature on medical points is limited. “

**Response**:

Thank you for the insightful comment. Some findings were restated to underscore their significance and operational implications. We also intentionally avoided broad recommendations that rely on political decisions, such as permitting humanitarian aid, and instead focused on practical, actionable steps for the medical community—such as participating in telemedicine.

The literature on medical points (MPs) is indeed scarce. However, in response to your valuable suggestion, we incorporated an example from another conflict setting (Syria) to better contextualize our findings and illustrate transferable strategies. Specifically, after the paragraph beginning with “Two-thirds of the MPs were hosted in tents and other temporary structures…”, we added the following:

*“Some practical lessons can be drawn from war-affected regions like Syria, where new health facilities were deliberately built away from town centers to minimize civilian casualties in case of airstrikes, and services were divided across separate locations to avoid total service disruption from a single attack (Haar et al., 2024). While such decentralization improved access and resilience under bombardment, it also complicated coordinated care, highlighting both the utility and limitations of MPs in conflict settings.”*

5. **Reviewer’s Comment:**

“It may be useful to speak to the blockade that may affect the influx of critical medications and equipment in the Gaza Strip.”

**Response:**
Thank you for this valuable suggestion. We have added a new paragraph to the Discussion section that directly addresses the role of the longstanding blockade and its escalation after October 7, 2023, in worsening medical shortages. The paragraph is as follows:

*“The blockade on Gaza—already in place for over 16 years—had severely under-resourced the health system even before the current war. Since October 7, 2023, the crisis has been compounded, turning the blockade into a critical barrier to medical response by restricting the sustained entry of essential medicines and equipment (WHO EMRO, 2024). Médecins Sans Frontières reported a continued inability to bring medical supplies into Gaza since April 2024, warning of severe shortages that left patients with burns, open fractures, and other traumatic injuries without basic painkillers, and forced surgical teams to operate without anesthetics (MSF Canada, 2024; MSF, 2024). The blockade has thus become a lethal determinant, accelerating the collapse of healthcare infrastructure and rendering even basic care unattainable.”*

This paragraph was added after the discussion of equipment shortages to maintain coherence and improve contextual interpretation of our findings. We have also included the relevant references

**Limitations**:

**Reviewer’s Comment:**

*“*This section can be expanded to include response rate, that the study only looked at MPs and its results may not be reflective of all medical structures in Gaza, among others.”

**Response:**
Thank you for this insightful suggestion. We have revised the Limitations section to address the response rate, the scope of the study, and an additional limitation regarding the use of self-reported data. We also included a brief justification for the potential bias due to nonresponse, noting the extraordinary conditions under which data collection took place.

**Revised Limitations Section (updated text):**

*“While our study provides valuable insights into the functioning of MPs in Gaza, its findings are limited by the cross-sectional design and the volatile and rapidly changing situation on the ground. The response rate was 63.6% (28 out of 44 identified MPs), possibly introducing response bias. However, this rate was achieved under active displacement and infrastructure destruction conditions. We noted that some non-responses were likely due to internet and electricity outages. During the study period, Gaza experienced severe communications disruptions – frequent internet blackouts and infrastructure damage often left communities cut off from communication [**29**]. We attempted follow-up calls where possible, but connectivity issues impeded several medical points from submitting the form. Furthermore, as the study focused exclusively on medical points, the findings may not be generalizable to other healthcare structures such as hospitals, field hospitals, or primary healthcare centers. Finally, although the study relied on self-reported data, responses were provided by healthcare workers directly involved in MP operations, offering firsthand and timely insight.”*

**Conclusion**:

1. **Reviewer’s Comment:**

Please specify “urgent action”.

**Response:**
Thank you for the comment. While we agree that general calls for humanitarian access are important, such recommendations often fall outside the scope of what the medical community can realistically act upon. Therefore, we revised the conclusion to specify urgent actions that are operationally feasible. The conclusion was revised to include the following sentence:

*“While the political actors are responsible for stopping this crisis, the medical and humanitarian community can contribute through immediate, actionable measures such as expanding telemedicine services, supporting remote training for Gaza-based providers, and offering psychosocial support to frontline healthcare staff.”*
