# Supplementary material for: Resilience amid chaos: The role of Gaza medical points
Source: PLOS Glob Public Health. 2025 Sep 17;5(9):e0005073. doi: 10.1371/journal.pgph.0005073 (PMC12443234; doi:10.1371/journal.pgph.0005073)
Supplement: Attachment — Submitted filename: Responses to Reviewer 2 Comments.docx [file pgph.0005073.s005.docx]

**Response to Reviewer 2 Comments**

We would like to clarify that the delay in responding to Reviewer 2’s comments was due to an oversight during our revision process. At the time, we were closely tracking and responding to comments from Reviewer 1, which we had mistakenly assumed to be the only set of reviewer comments. As a result, we unintentionally missed the detailed feedback provided in the second review. We apologize for this error and the time and inconvenience it may have caused. Upon recognizing the omission, we undertook a thorough re-examination of the manuscript in light of Reviewer 2’s remarks and have now addressed each point with the attention it rightfully deserves.

In addition, we sincerely thank Reviewer 2 for their thoughtful and constructive feedback, which has substantially strengthened our manuscript. We are particularly grateful for their generous remark that this work represents “a huge contribution to the literature on conflict and health.” Our research team in Gaza has invested significant time and effort under extremely challenging conditions to bring this study to light, and we are deeply encouraged by the reviewer’s recognition.

Below, we provide point-by-point responses to each of the reviewer’s comments, and we hope the revisions meet their expectations.

**Introduction:**

1. **Reviewer’s Comment:** “Lines 65–68: Does the exacerbation of strain refer to the effect of the frequent 5 wars and the siege? If so, please make this clear here.”
2. **Reviewer’s Comment:** “Line 69: Suggest rewording to clarify this, something to the effect of ‘The ongoing military assault on Gaza that began in 2023…’.”

**Response:** We thank the reviewer for these insightful observations. Their understanding is correct, and the revised text now reads:

“…This strain was **further exacerbated by repeated military attacks (2008–09, 2012, 2014, and 2021), sustained siege, and funding shortages, which have collectively restricted access to medical supplies and undermined healthcare services in Gaza even before the current escalation.**

**Building on this, the ongoing military assault on Gaza that began in 2023** has had devastating effects on the district’s medical infrastructure, resources, and personnel, pushing the healthcare system to the brink of collapse as documented by the Office of the High Commissioner for Human Rights (OHCHR) [2].

***********************************************************

3. **Reviewer’s Comment:** “Line 72 – physical assault on who? Does this paragraph refer specifically to attacks on health workers or more generally? If about healthcare, please make this clear.”

**Response:** We appreciate the reviewer pointing out this ambiguity. The paragraph indeed refers to attacks specifically on healthcare workers. Accordingly, the revised sentence now reads:

“**This assault has involved direct violence against healthcare workers, disruption of service delivery, and the criminalization, arrest, and detention of both patients and staff.”**

**********************************************************

4. **Reviewer’s Comment:** “Line 75: suggest changing ‘divided by’ to ‘including’.”

**Response:** Thank you for this suggestion. While addressing this, we noticed an apparent discrepancy in the reported figures, which prompted us to re-examine the original source. Upon review, we found that the WHO Health Attacks Dashboard had been updated since our initial submission. Accordingly, we updated the statistics in this and the following sentence to reflect the most recent available data (as of May 2025), ensuring consistency and accuracy across the revised text. The reference has been updated accordingly, and an additional reference has been incorporated. The text now reads:

“**As of May 22, 2025, 720 attacks on healthcare had been reported in Gaza, resulting in 917 deaths, around 1,000 injuries, and the detention of 300 health workers and 70 patients** [**Add Ref 1**].

**In the same month, 18 hospitals were partially functional across Gaza, with 18 of 36 hospitals completely out of service. Additionally, 8 field hospitals were operational, including five fully and three partially functional hospitals in various locations. At the same time, 39% of primary health care centers were functional (most of them partially), and only 33% of UNRWA health centers remained operational** [**updated Ref 2**].

**References:**

**Add Ref 1 (ref 3 in the final version): Health Cluster’s Unified Dashboard [Internet]. 2025 [cited 2025 May 22]. Available from:**

[**https://app.powerbi.com/view?r=eyJrIjoiODAxNTYzMDYtMjQ3YS00OTMzLTkxMWQtOTU1NWEwMzE5NTMwIiwidCI6ImY2MTBjMGI3LWJkMjQtNGIzOS04MTBiLTNkYzI4MGFmYjU5MCIsImMiOjh9**](https://app.powerbi.com/view?r=eyJrIjoiODAxNTYzMDYtMjQ3YS00OTMzLTkxMWQtOTU1NWEwMzE5NTMwIiwidCI6ImY2MTBjMGI3LWJkMjQtNGIzOS04MTBiLTNkYzI4MGFmYjU5MCIsImMiOjh9)

**Updated Ref 1 (ref 4 in the final version): United Nations Office for the Coordination of Humanitarian Affairs (OCHA). Reported impact snapshot: Gaza Strip, 28 May 2025 [Internet]. Geneva: OCHA; 2025 [cited 2025 Jun 10]. Available from:** [**https://www.ochaopt.org/content/reported-impact-snapshot-gaza-strip-28-may-2025**](https://www.ochaopt.org/content/reported-impact-snapshot-gaza-strip-28-may-2025)

*********************************************

5. **Reviewer’s Comment:** “Lines 84–87: suggest to also state that many people have been displaced multiple times.”
   **Response:** We agree with this valuable note and have added a statement highlighting repeated displacements and their consequences. In addition, we added a reference for the number of 1.9 million people displaced. The revised text now reads:

"With ongoing evacuation orders issued by Israeli forces and repeated waves of displacement, approximately 1.9 million people have been displaced **by May 2025, many displaced multiple times [Add Ref 2]—intensifying their vulnerability, disrupting access to consistent healthcare, and complicating the management of pre-existing conditions. As of May 2024, 81% of Gaza remained under active Israeli evacuation orders.**^4^"

**Add Ref 2 (ref 5 in the final version)**: **United Nations Relief and Works Agency for Palestine Refugees in the Near East (UNRWA).** UNRWA situation report #169 on the situation in the Gaza Strip and the West Bank, including East Jerusalem [Internet]. UNRWA; 2025 May 27 [cited 2025 Jun 10]. Available from:

<https://www.unrwa.org/resources/reports/unrwa-situation-report-169-situation-gaza-strip-and-west-bank-including-east-jerusalem>

****************************************

6. **Reviewer’s Comment:** “*Please review the links in the references, some of these are not working.*”
   **Response:** We have rechecked all the reference links and found them to be functional. If the reviewer is still encountering an issue, we kindly ask them to specify which link is not working so that we can address it accordingly. Please note that certain links become intermittently disrupted after citation formatting. For example, reference [2] originally directed to “*Thematic report: Attacks on hospitals during the escalation of hostilities in Gaza, 7 October 2023–30 June 2024*”, but during the review stage, it occasionally returned a “Page Not Found” error. To resolve this, we updated the link to the ReliefWeb version, where the report is consistently accessible under the “Download Report” section. However, we noticed that clicking the hyperlinked word “report” in the text may still inconsistently redirect to either the correct document or an error page, depending on the session or browser.

*****************************************

*****************************************

**Methodology:**

1. **Reviewer’s Comment:** “Please describe the sampling method. How many MPs exist in Gaza (is it 44)? Was every single medical point contacted via SMS?”

**Response:** We have added a new subsection “**Sampling frame and recruitment**” at the beginning of Methods. It now states that the Ministry of Health (MoH) listed 44 accredited medical points (MPs) across Gaza. All 44 were contacted via SMS and received up to three reminder messages. The full paragraph now reads:

“**Using the MoH master roster** **(version 18 Sep 2024), all 44 functioning MPs were enumerated. Study links were distributed by SMS to the telephone number registered for each MP and reminders were sent up to 3 times after initial contact.**”

2. **Reviewer’s Comment:** “First paragraph (lines 124–138): Please move the description of the sampling to be placed before the description of data collection.”

**Response:** Done. The paragraph above now precedes the description of the Google-Form and data collection.

3. **Reviewer’s Comment:** “Lines 130–131: The sentence about failure to respond from lack of internet… this should also be described in the results, not the methods.”

**Response:** We have moved the discussion of non-response due to connectivity issues from the Methods to the Results section. In Methods we now simply note outreach attempts; in Results we discuss response rates. The beginning of the Results section now reads:

“The response rate among contacted medical points in Gaza was 28/44 (63.6%). **In many situations, failure to respond was due to the lack of internet connectivity, as reported by medical teams during follow-up phone calls. Some respondents had to move to another area to fill the survey as they had no connection at their locations.**

A comprehensive analysis was conducted on the data from responding medical points….”

4. **Reviewer’s Comment:**

Variables and measures: This reads as if the facilities were assessed, but the design is limited to self-report by the MP staff. It is important to make this distinction.

**Response:**

Thank you for this valuable comment. Accordingly, self-report clarified in the opening sentence:

**“All items were self-completed by a senior MP clinician; no external validation visit was possible”.**

5. **Reviewer’s comment:** Point 1 is clear. Point 2 suggests evaluation of capacity – this should be rephrased to something like ‘MPs were asked about their capacity to…’.

Done. Point 2 now reads:

“**MP staff were asked about** their capacity to deliver emergency care, primary care, maternity care and chronic disease management”.

6. **Reviewer’s comment:** Point 3 should be clarified. Were participants asked about medication supply for the 10 categories? If so, please reword to make this clear

**Response**: Done. Point 3 now reads:

**“Participants rated the availability of medications in 10 therapeutic categories**, including…**”**

7. **Reviewer’s comment:** Point 4 is clear, but it would be improved if the second sentence were reworded to something such as “Participants were asked about their working hours, psychosocial support, and training opportunities.”

**Response:** Done. Point 4 second sentence now reads: “**Participants were asked about their working hours, psychosocial support and training opportunities**.”

8. **Reviewer’s Comment:** “Please specify the time frame that the MPs were surveyed about. (Which period? Time frame for supply/stock questions?)”

**Response:**

We thank the reviewer for this important observation. While the questionnaire did not specify a time frame, responses are understood to reflect the situation during the data collection period (October–December 2024). This clarification has now been added at the end of “Variables and Measures” subsection within the Methods section of the revised manuscript as follows:

“**Responses are understood to reflect the situation** **during the data collection period (October–December 2024), as no specific reference period was indicated in the questionnaire.**”

9. **Reviewer’s Comment:** “Did the survey ask about how services were advertised to the community? For example, if trauma services were provided, how would patients know they could go to the MP rather than a hospital?”
   **Response:**

We thank the reviewer for this insightful comment. The survey did not include items on how services were advertised or communicated to the community. This has now been acknowledged in the Discussion section as follows:

“In parallel, clinical support from external professionals must be not only technically competent but also culturally and linguistically aligned with Gaza’s population. **Our survey did not assess how services were communicated or advertised to the local community, which may affect utilization patterns and awareness of available care.**

Despite these challenges, such initiatives represent a crucial step toward alleviating the healthcare crisis in Gaza.”

10. **Reviewer’s Comment:** Could you please share the survey questions in supplementary materials, so readers can understand how the data were collected? I appreciate this would require translation to English but it would be very useful.

**Response**:

We appreciate the reviewer’s suggestion. The survey was originally distributed in English and has now been included in the supplementary materials to enhance transparency and allow readers to better understand the data collection process.

**11. Reviewer’s Comment:** There is some repetition in the first and second sentences of this section.

**Response:**

The repetition has been addressed in a prior revision, and the first two sentences of the section now read as follows:
“**Using the MoH master roster, all 44 functioning MPs were enumerated. Study links were distributed by SMS to the telephone number registered for each MP and reminders were sent up to 3 times after initial contact.**”

The sentence describing the study design and data collection now appears separately to improve clarity.

********************************************

********************************************

**Results:**

1. **Reviewer’s Comment:** “Line 172 – suggest rewording to “The dataset included information from 28 medical points….”

**Response:** This comment has already been accommodated in a previous revision. The relevant sentences now read:

**“The response rate among contacted medical points in Gaza was 28/44 (63.6%). In many situations, failure to respond was due to the lack of internet connectivity…A comprehensive analysis was conducted on the data from responding medical points.”**

2. **Reviewer’s Comment:** “Line 182: Were workers physically harmed while working in the medical point? Please specify this.”

**Response:** The survey did not ask about the specific location of injury or death. We have now clarified this in the revised manuscript as follows:

3. “Four out of 28 medical points (14.3%) reported at least one staff member being injured or arrested during the period.  One worker was killed in an airstrike. **Place of injury or death was not specified in the survey**.”
4. **Reviewer’s Comment:** “Lines 190–191: Were there strips for the glucometers? It is important to note that there is a supply requirement for these also, many public health professionals won’t be aware of that.

**Response:** We thank the reviewer for this valuable comment. The survey item explicitly referred to a “working glucometer with strips,” indicating that both the device and strips were available. We have revised the sentence in the manuscript accordingly. It now reads:

“Essential tools, including pulse oximeters (n=11) and **working** glucometers **with strips** (n=11), were available in less than half of the points.”

5. **Reviewer’s Comment:** “Line 192: Suggest removing ‘the fact’ – this is based on participant report, and should be reported as such.”

**Response:** We thank the reviewer for this suggestion. The phrase has been revised to reflect that this is based on participant report. The sentence now reads:

“**Although most MPs reported receiving** cases of trauma regularly, suture kits were not available in one-third (n=9, 32.1%).”

6. **Reviewer’s Comment:** “Did participants report on what they did when supplies ran out (e.g. suture kits, disinfectant or bandaging)?”

**Response:** Our survey did not include follow-up questions on actions taken after stockouts. Accordingly, the following sentence was added to the Discussion after talking about supply limitations:

”The severe shortage of critical medical equipment, including oxygen supplies, portable ultrasound machines, **suture kits** and sterilization materials, severely limits the capacity of medical points to deliver adequate care, particularly for trauma and critical patients. With the rising number of such cases during the assault on Gaza, the absence of these supplies is **likely** contributing to preventable deaths. **While respondents detailed the availability of critical supplies, the survey did not examine how staff coped during stockouts—an important operational gap that warrants future qualitative investigation.**”

7. **Reviewer’s Comment:** Please report on the numbers and training of staff that provide services in the MPs. Description of staffing as well as the kinds of services offered (lines 219-226) should be moved to early in this section, so the reader can understand the available resources for staffing as well as equipment.

**Response:**

The Results section has been restructured in response to this comment. We now report the total number of staff (doctors, nurses, paramedics, pharmacists, and support staff), as well as the median, interquartile range, and per-unit distributions. Additionally, information regarding staff training, availability of psychological support, and salary regularity, in addition to the description of the services offered by the MPs (e.g., primary care, trauma care, maternity services, mental health services) have all been relocated to precede the equipment and drug availability subsection. These changes aim to provide readers with a clearer understanding of the human resources available before discussing material resources. The first paragraphs of the revised Results section now read (additions in bold, relocated content in italics).:

**“Results**

The response rate among contacted…

A comprehensive analysis was conducted on medical point…

Out of all surveyed medical points, 10(36%) were explicitly identified as part of the Ministry of Health’s operational network. …Two (7.1%) MPs were attacked with significant infrastructural damage.

*Staffing levels varied by unit (Fig. 2).* **Across the 28 responding medical points (MPs) we documented 68 doctors, 60 nurses, 28 paramedics, 30 pharmacists, and 62 other staff, for an overall workforce of 248 personnel (median 7 per MP, IQR 5–10, range 2–20). Medians by cadre were 2 doctors, 2 nurses, 0 paramedics, 1 pharmacist, and 2 support staff per MP, and three-quarters of units operated with no more than two physicians while 61 % had no paramedic on site.** *As for training, 67.9% of workers (n=19) reported receiving no training since joining their units. Psychological support of the staff was inadequate, with two-thirds reporting no support (n=12, 42.9%) or minimal support (n=8, 28.6%). Salaries were paid on time in 12 units (42.9%), with four units (14.3%) experiencing minor delays, while the remaining units reported inconsistent payments, including two units where no salaries were received.*

*Most units provided primary care (n=27, 96.4%), pediatric care (n=25, 89.3%), and care for chronic diseases (n=25, 89.3%). Less than half of the units provided maternity care (n=11, 39%) or emergency trauma care (n=13, 46%). Dermatological, orthopedic, and ENT services were available in only one MP. Mental health services were available in only one other MP, as well. Some of the MPs hosted special programs for health education (n=16, 57.1%), sexual and reproductive health services (n=6, 21.4%), vaccination campaigns (n=11, 39.3%), nutrition programs (n=14, 50.0%), and prenatal care (n=5, 17.9%).*

Regarding facility and equipment availability, the analysis found that MPs…”

Additionally, a clarification was added to the “Data Analysis” section:

“Quantitative data were analyzed using descriptive statistics, including frequencies, medians, ranges, and interquartile ranges. Data was analyzed using R program (version 4.4.1). Likert and ggplot2 were used to make figures. **For workforce analysis, the non-numeric response “More than 5” was recoded as 6 to allow inclusion in staff totals and medians.**”

A corresponding note was also added to the Limitations section::

“**Regarding data analysis, some staffing responses were non-numeric (“More than 5”) and were conservatively recoded as 6, which may slightly underestimate total workforce counts.**”

8. **Reviewer’s Comment:** “Please rework the drug supply paragraph or share the Likert scale so the reported numbers can be clearly understood. Also, the findings show very limited cardiovascular medication availability – I appreciate there was better availability, but it seems like a bit generous to say it was “the best”. I suggest to word it slightly differently: “Cardiovascular drugs, including hypertensives, had slightly better availability, with 18 MPs reporting access to…”

**Response:** We thank the reviewer for this thoughtful comment. This point was previously addressed in the Methods section in response to Reviewer 1; however, we have now revised the clarification to be more specific. The revised text under the **“Drug Availability”** subsection of the **Methods** section now explicitly states the meaning of each point on the Likert scale, as follows:

“Drug Availability: **Participants rated the availability of medications in 10 therapeutic categories, including antibiotics, antipyretics, antihypertensives, and insulin, using a five-point scale: 1 = not available (no drug in category available), 2 = available (1 or 2 medications), 3 = half of needed medications available, 4 = most medications available (1 or 2 missing), and 5 = all needed medications available.”**

We also modified the relevant paragraph of the **Results** section as follows**:**

**As for medication availability, the mean availability score for antibiotics was** 1.8 (SD = 1.4), with 12 **MPs** (42.9%) reporting no availability. Analgesics **had a mean availability of 2.2 (SD = 1.4), with 13 points (46.4%) scoring 3 or higher. Cardiovascular drugs, including antihypertensives, were relatively more available,** with 18 MPs reporting access to half or more of the needed medications. Drugs that showed the worst shortages included respiratory medications, psychiatric medications, antiepileptic drugs and cancer drugs (Fig 2). Antidiabetic medications had limited availability with only 2 (7.1%) of the MPs reporting any insulin supply.

9. **Reviewer’s Comment:** Please report number (N) and percent for all statistics shared. At present it is variably reported, sometimes just a number, sometimes both number and percent.

**Response:** We have standardized our reporting. All results now present both the count (n) and percentage (%) consistently. For example:

"MPs were hosted in tents and other temporary structures in 19 (67.9%). Electricity availability varied significantly, **with 15 MPs (54%)** reporting it as unavailable,…”

10. **Reviewer’s Comment:** “Line 222: Were the mental health services provided by trained professionals? What kind (doctor, psychologist, paraprofessional)? How many MPs offered mental health services? Were participants asked who provided psychological support, or if there was a formal service for health workers? This is very important.”

**Response:**

We appreciate the reviewer’s thoughtful questions regarding the provision of mental health services. Mental health service availability was assessed through a drop-list question (Q3.1) asking respondents to indicate which services were offered at their unit. “Mental health services” was included as a response option, but follow-up questions on the provider’s qualifications or type (e.g., psychologist, social worker, or volunteer) were not included.

We respectfully remind the reviewer, as they themselves noted, that this study was conducted under exceptionally difficult conditions amid bombardment, displacement, and infrastructure collapse. The survey was intentionally kept brief to reduce burden and improve response rates. While we recognize the importance of more in-depth data, such constraints limited our ability to include detailed follow-up. We agree this remains an important area for future investigation.

11. **Reviewer’s Comment:** Line 240 – please specify how long the MP was functioning with staff that were not receiving salary.

**Response:** The survey asked whether staff were being paid for their work using predefined response categories (Q4.10): “Always paid on time,” “Most of the time/minor delays,” “Sometimes/some delays,” “Rarely/major delays,” and “Not at all.” It did not include a follow-up question regarding the duration for which staff were unpaid. As such, we are unable to report how long each unit functioned without salary support.

*****************************************************

*****************************************************

**Discussion
1. Reviewer’s Comment:** The discussion focuses heavily on telemedicine but is much weaker on addressing the systemic issues. There are some studies on telemedicine now which help to describe the uses as well as challenges and risks – and it is only palliative because it doesn’t stop the underlying problems, such as limited staff, supply disruptions, lack of equipment, attacks on health care. The discussion would be much stronger if it also directly addressed some of these factors.

**Response**:

We thank the reviewer for their valuable observation. In fact, we wish to clarify our approach. In this paper, we deliberately emphasized actionable field-level recommendations rather than reiterating longstanding calls that require political resolution. While many systemic deficiencies have been acknowledged for years, repeated appeals for comprehensive policy reform have yielded little change on the ground. Our aim was to highlight interventions that could be operationalized even in the face of political paralysis.

That said, we recognize the value in briefly addressing key systemic remedies, and have accordingly added the following paragraph right before talking about telemedicine:

“**Given the long-standing political paralysis surrounding Gaza’s health crisis—despite repeated international appeals—our recommendations focus on practical measures that remain viable amid sustained inaction. Implementing a locally managed, transparent supply-tracking system could help prioritize critical shortages and enhance distribution efficiency. In parallel, task-shifting programs to train non-specialist staff in basic emergency and chronic care may partially alleviate the burden created by the ongoing loss and exhaustion of qualified personnel. These steps cannot address the structural roots of the crisis; without reliable humanitarian access to restore essential supplies, all solutions remain partial and palliative—insufficient to counter the obstruction of life-saving aid. Nonetheless, they represent actionable pathways to sustain medical point operations under extreme constraints**.”

****************************************************

**2. Reviewer’s Comment:** Line 256- is the jaundice seen in kids from hepatitis? Or could it also be from non-infectious causes?

**3. Reviewer’s Comment:** Lines 255-256: please provide a citation for this sentence.

**Response**:
In response to the reviewer's query regarding the etiology of jaundice among children under five in our study, it's important to note that while our data collection did not include laboratory diagnostics to ascertain specific causes, the timing, epidemiological pattern, clinical context and environmental conditions suggest that the observed jaundice is predominantly attributable to infectious hepatitis, particularly hepatitis A. This is further emphasized by the report of UNRWA “Gaza’s displaced people face a new peril: Hepatitis A outbreak”

Accordingly, we modified the sentence as follows:

“This shortage is occurring in a context where Gaza is already experiencing a critical health crisis among children under 5 years, characterized by a significant rise in infectious diseases, including acute respiratory infections, diarrhea, scabies, and **hepatitis A [Add Ref 3, Add Ref 4]**.”

The first reference talks about the alarming rise in the mentioned infectious diseases among children younger than 5 years in Gaza, and the second reference specifically mentions the hepatitis A outbreak.

**Add Ref 3 (ref 18 in the final version):** Taha AM, Mahmoud H, Nada SA, Abuzerr S. Controlling the alarming rise in infectious diseases among children younger than 5 years in Gaza during the war. Lancet Infect Dis. 2024 Apr;24(4):e211. doi: 10.1016/S1473-3099(24)00067-7. Epub 2024 Jan 30. PMID: 38307097.

**Add Ref 4 (ref 19 in the final version):** United Nations Relief and Works Agency for Palestine Refugees in the Near East (UNRWA). Gaza’s displaced people face a new peril: Hepatitis A outbreak [Internet]. 2024 Mar 25 [cited 2025 June 10]. Available from: <https://www.unrwa.org/newsroom/features/gaza-displaced-people-face-new-peril-hepatitis-outbreak>

***************************

**4. Reviewer’s comment:** Lines 263-274: Recommendations for mental health services in humanitarian settings place emphasis on access to basic needs, strengthening community supports, and then smaller non-specialist services, and lastly, medications and specialist support. The focus here on medications implies the reverse and undermines the paper, as it gives the impression that mental health services are focused specifically on prescribing medication, when this is not standard of care even in peaceful and well-resourced settings. It would help to provide some context for why there is a focus on medications – was there a population that was already receiving medications prior to this war, and who are now no longer able to access their medicines? Or is there another reason for the emphasis on medication?

**Response:** This is a very important corrective, and we thank the reviewer for highlighting the need to frame our findings on psychiatric medication shortages within the established Mental Health and Psychosocial Support (MHPSS) pyramid. Accordingly, the paragraph has been substantially revised to first acknowledge the foundational layers of the MHPSS pyramid and then to put our findings in context. It now reads:

"The severe shortage of psychiatric medications reported here (>90% limited or unavailable) is alarming, particularly given the previously reported prevalence of 22% for conditions such as depression, anxiety, post-traumatic stress disorder, bipolar disorder, or schizophrenia among individuals affected by war or other conflicts.^9^ **International guidelines recommend various activities for providing mental health and psychosocial support (MHPSS) during emergencies, ranging from community self-help and communications to psychological first aid and clinical mental health care.** **^9^** **A recent review offered insights for improving mental health and psychosocial support (MHPSS) for Gaza’s populations [Add Ref 5]. Our findings on critical psychiatric medication shortages are particularly alarming because they signify an acute disruption of essential treatment for individuals already affected or at high risk of developing mental health conditions, especially within a context of pervasive trauma and loss.** This **medication crisis** is further compounded by the extremely limited availability of mental health services in medical points as shown in our study, with only 7% offering such care, **leaving a profound unmet need for mental health care across key recommended intervention layers.**

A similar concern arises from the severe shortage of antiepileptic drugs,…."

**Add ref 5 (ref 21 in the final version):** Aqtam I. A narrative review of mental health and psychosocial impact of the war in Gaza. East Mediterr Health J. 2025 Mar 3;31(2):89-96. doi: 10.26719/2025.31.2.89. PMID: 40116261

*****************************************

**5. Reviewer’s comment**: Lines 301-302: The study design does not allow for causal attribution. This statement should be amended, to “likely contributing to preventable deaths.” If there was an assessment of mortality, this should be reported in the results and discussed in the discussion.

**Response:** We agree with the reviewer and the statement has been amended as suggested:

“With the rising number of such cases during the assault on Gaza, the absence of these supplies is **likely** contributing to preventable deaths.”

******************************************

6. **Reviewer’s comment**: Lines 307-312: This paragraph on telemedicine should be expanded further. Telemedicine is useful as a palliative measure when other solutions to improve resources are not possible. There are barriers identified in this review – only 21.4% (n=6) had uninterrupted internet access and 35.7% (n=10) had no or rare access. So telemedicine will not work well for a large proportion of the MPs unless internet access improves.

Also, telemedicine could also be used to provide mental health support for health workers, as well as the general population of Gaza.

**Response:** In response to this and the following three comments regarding the telemedicine paragraphs, we have rewritten the entire section to incorporate the reviewer’s valuable suggestions. Kindly refer to the revised section, where all changes are shown in bold following the comments.

7. **Reviewer’s comment**:

Lines 313-323: were groups providing clinical services over WhatsApp? Or how was WhatsApp used? Please note that this violates all data protection laws and can place people in danger to both patients and health workers through health and location information becoming available and possible to intercept. The ICRC protection database was hacked in late 2022 – and that was a database that had significant protections put into place because it held sensitive information. (https://www.icrc.org/en/document/icrc-cyber-attack-analysis)
**Response: in the revised section below.**

8. **Reviewer’s comment**:

Lines 313-323: please provide citations for the different organizations and programs referred to here and evidence for their success.

**Response: in the revised section below.**

Please note that we cited the website of Gxza Health, where the “Achievements” section highlights the same accomplishments referenced in our manuscript. In addition, the section titled “How does our service work?” explicitly states: “The provider is notified about their patient case, and reaches out to the patient via WhatsApp.” We also cited the Lancet article titled *Trauma care supported through a global telemedicine initiative during the 2023–24 military assault on the Gaza Strip, occupied Palestinian territory: a case series*, which notes: “An additional inadvertent success from the group chat was the vast network it facilitated, connecting a range of stakeholders including health-care professionals, non-governmental organisation leaders, humanitarian aid workers, and media-affiliated individuals. This allowed for multiple press releases, as well as on-the-ground coordination for aid supply and medical evacuations, including through initiatives such as Gxza Health.”

As for “Seeds of Humanity” we had it in the original text based on the experience of the paper authors in Gaza. The only citation we found was about its accomplishments in the first 15 days of launching its initiative, which was in Arabic:

<https://www.linkedin.com/posts/seed-tr_%D9%86%D8%AA%D8%A7%D8%A6%D8%AC-%D9%85%D8%A8%D8%A7%D8%AF%D8%B1%D8%A9-%D8%A7%D9%84%D8%B9%D9%84%D8%A7%D8%AC-%D8%B9%D9%86-%D8%A8%D8%B9%D8%AF-%D9%84%D8%A3%D9%87%D8%A7%D9%84%D9%8A-%D8%BA%D8%B2%D8%A9-%D8%A7%D9%84%D8%AA%D9%8A-activity-7167760221401006080-TjZo/?originalSubdomain=ae>

Accordingly, we removed it from the text since we were not able to find an acceptable reference that testifies for its success

9. **Reviewer’s comment**: Lines 324-329 – please see my earlier note about WhatsApp. I appreciate this is handy and readily available but there are very real dangers to both patients and health workers with its use. These should be acknowledged, at the least. There is a need for readily available, secure and appropriate platforms for telemedicine as well as health workers outside of Gaza who understand the context and can provide telemedicine support.

**Response: in the revised section below.**

**Revised Section (addressing all four reviewer comments on the telemedicine section):
*All modifications are presented in bold within the text below.***

“Healthcare professionals (HCPs) outside Gaza can play a vital role in mitigating the catastrophic healthcare situation through telemedicine. By offering virtual consultations, diagnostic support, and mental health services, telemedicine can partially overcome physical barriers to healthcare in war zones.^14^ It holds promise not only to address the mental health needs of Gaza’s general population but also to support its overburdened healthcare staff**—especially given that two-thirds reported receiving no or minimal psychosocial support.**

Several organizations and volunteer teams have established online platforms, such as WhatsApp groups (WhatsApp, Meta; Menlo Park, CA, USA)**. For example, Gxza Health reports providing thousands of teleconsultations, psychosocial support, and coordinating medication delivery through WhatsApp-based outreach (Add Ref 6, ref 15 in original version).** One study on the use of telemedicine at Nasser Hospital in Gaza demonstrates how **telemedicine support has emerged as a vital lifeline for health-care workers on the ground, allowing for the provision of multispecialty advice for complex patient cases**.^34^ These findings underscore the potential for similar telemedicine approaches to be adapted and expanded within Gaza, including medical points.

**However, while these efforts represent palliative attempts to bridge critical care gaps where formal systems are severely strained, they face several challenges. First,** **their effectiveness is profoundly hampered by Gaza’s intermittent internet and electricity. Our findings indicate only 21.4% (n=6) of surveyed medical points (MPs) reported full internet availability, and 35.7% (n=10) had no or rare access, with consistent electricity also being a major issue.** Additionally, public engagement has been slow due to the novelty of these initiatives and weak advertising. **Moreover, the use of commercial messaging platforms for clinical purposes raises critical data protection, privacy, and security concerns. Although WhatsApp offers end-to-end encryption, it is not designed or certified for managing sensitive health data in conflict zones. Health and location information of both patients and health workers may be exposed to hostile interception. The 2022 cyberattack on the International Committee of the Red Cross's (ICRC) protected database (Add Ref 7) highlights the risks of relying on unsecured digital systems, and the urgent need for secure, low-bandwidth, contextually appropriate telemedicine platforms designed for conflict settings. In parallel, clinical support from external professionals must be not only technically competent but also culturally and linguistically aligned with Gaza’s population. Our survey did not assess how services were communicated or advertised to the local community, which may affect utilization patterns and awareness of available care.**

Despite these challenges, **increasing positive feedback from beneficiaries has gradually improved participation, and these** initiatives **now** represent a crucial step toward alleviating the healthcare crisis in Gaza.”

**Add Ref 6 (ref 32 in final version):** Gaza Health: Our services and achievements [Internet]. Gaza: Gaza Health; [cited 2025 Jun 10]. Available from: <https://gxzahealth.org/>

**Add Ref 7 (ref 34 in final version):** International Committee of the Red Cross. Cyber-attack on ICRC: What we know [Internet]. Geneva: ICRC; 2022 [cited 2025 Jun 10]. Available from: <https://www.icrc.org/en/document/cyber-attack-icrc-what-we-know>

*********************************************
**10. Reviewer’s comment**:

Limitations – I think it is important to recognize that this study was carried out in spite of enormous challenges. The fact that it was even completed is a testament to the feasibility of doing research in areas experiencing high intensity conflict. Demonstrating this feasibility – even if it is simply a cross-sectional study – is still a huge contribution to the literature on conflict and health.

**Response**:

We sincerely thank the reviewer for recognizing this aspect of the study. In response, we have added the following paragraph at the end of the Limitations section:

**“Importantly, the successful completion of this study during ongoing military assault and systemic infrastructure collapse underscores the feasibility of conducting field research in extreme conditions. Despite being a cross-sectional study, it offers a rare, practice-based contribution to the conflict-health literature by demonstrating that meaningful data collection is possible even under severe hardship.”**

****************************************************
