# Supplementary material for: Resilience amid chaos: The role of Gaza medical points
Source: PLOS Glob Public Health. 2025 Sep 17;5(9):e0005073. doi: 10.1371/journal.pgph.0005073 (PMC12443234; doi:10.1371/journal.pgph.0005073)
Supplement: Attachment — Submitted filename: renamed_bc66a.docx [file pgph.0005073.s006.docx]

**Reviewer #1:** Thank you for addressing the comments so thoroughly. My only comment is for figure 1 where one of the medical facilities appears to not be on land; please adjust this.

**Response:** Thank you for your valuable observation regarding Figure 1, where one of the medical facilities appeared to be located off land. We have carefully reviewed the figure and corrected the placement issue. The revised version has been attached under the title “Figure 1 adjusted per reviewer’s comment.”

We appreciate your attention to detail and your constructive feedback, which has improved the clarity and accuracy of our manuscript.

Regards,
Prof. Eyad Qunaibi

On behalf of the authors
